# Effects of posture change on nasal patency

**DOI:** 10.1016/S1808-8694(15)31203-9

**Published:** 2015-10-20

**Authors:** Renato Roithmann, Pedro Demeneghi, Roberta Faggiano, Alexandre Cury

**Affiliations:** 1 Joint Professor of Otorhinolaryngology, Universidade Luterana do Brasil, Joint Professor of Anatomy, Federal University of RS, Professor, Post-graduation in Medicine: General Medicine, UFRGS, Otorhinolaryngologist.; 2 Intern, Service of Otorhinolaryngology, Universidade Luterana do Brasil.; 3 Resident Physician, Former Monitor in Otorhinolaryngology.; 4 Resident in Otorhinolaryngology, Universidade Luterana do Brasil.

**Keywords:** posture, acoustic rhinometry, nasal patency, nasal obstruction

## Abstract

Nasal obstruction when lying down frequently brings patients to the otolaryngologic clinic. There are several explanations for the problem. The nasal mucosa reaction to venous changes that alter local blood flow, secondary to compression of the neck veins or hydrostatic pressures, is the most accepted explanation. Acoustic rhinometry is a new non-invasive technique to assess nasal patency. **Aim:** The purpose of this study was to assess the effect of posture change from sitting to supine position applying acoustic rhinometry. **Study design:** clinical prospective. **Material and method:** 10 volunteers with no nasal disorders, aged 19 to 30 years old, and 10 volunteers with symptoms of rhinitis, aged 18 to 27 years old, were selected for the study. Nasal sensation was tested by means of a visual analogue scale. Nasal area and volume were assessed by acoustic rhinometry in the following positions: seated and 15 minutes after lying down. **Results:** Both groups showed significant nasal obstruction on the visual analogue scale and on acoustic rhinometry. The perception of nasal obstruction was significantly higher in subjects with rhinitis symptoms compared to normal. **Conclusion:** We conclude that the effect of posture change from sitting to supine position produces a decrease in nasal cross-sectional area and volume in both normal and in subjects with symptoms of rhinitis. However, the impact on the perception of nasal obstruction induced by lying down seems to be higher in subjects with symptoms of rhinitis.

## INTRODUCTION

The sensation of nasal obstruction upon lying down is a commonly related experience by patients in daily ENT care. Many explanations are advocated for the perception of this phenomenon in specific people 1, 2, 3. Among them we can refer to:

1. Possibility of a feedback loop processed by the central nervous system (CNS), alternating the information of patency of nasal cavity to modify the vascular ingurgitation of the structure;
2. Venous pressures that affect the blood content of the nasal mucosa differently on one side than on the other, by compression of neck vein, rather than hydrostatic differences;
3. The induction in lateral position of active reflex responses that determine the resistances to airflow of each nasal cavity;
4. The pressure to the lateral of trunk and limbs that follow posture asymmetry in decubitus and consequently the indication of nasal congestion on the side of the higher pressure and contralateral decongestion;
5. The hydrostatic increase of venous pressure and relaxation of vasomotor tone;
6. The response of nasal mucosa for both local and systemic conditions (hydrostatic), probably induced by vascular and cutaneous reflex.

Most of the studies that objectively analyzed the influence of posture affections on nasal breathing used the rhinomanometric method to determine patency of nasal airway 4, 5. Variations of nasal patency caused by the changes in posture were perfectly assessed by rhinomanometry ^2^. The Rhinomanometry calculates the resistance of transnasal airway, or more simply, how difficult it is to breathe through the nose 2, 4. The rate takes into consideration consecutive measurements of airflow and transnasal pressure (Rn = DP/V, where Rn = nasal resistance, DP = difference of atmospheric pressure and rhinopharynx, and V = transnasal airflow).

Comparative measurements of nasal airway resistance in different positions of the body were described for the first time in 1964 by Rundcrantz^6^ in a series of rhinitis patients. Later, Rundcrantz^5^ observed that total nasal resistance was higher in supine position than in seated position also in normal subjects. Hasegawa^7^ also showed changes in airway resistance as a result of the change in posture and stated that in dorsal position the airflow resistance on the nasal congested side was higher than in vertical position.

Cole & Haight^2^ calculated the unilateral and total transnasal air resistance in different body position and found that lateral position reduced nasal patency on the side that was turned down and increased it on the contralateral side. The finding of relevance was that total nasal resistance (right more than left) was minimally reduced in the study.

In 1989, Hilberg et al.^8^ described a new technique of assessment of nasal patency and named it acoustic rhinometry. These authors tested for the first time the acoustic reflex technique, only used to assess tracheal and lower airways diameter in the nose, and they described the graph in the area as a result of nostril distance in the normal cavity.

Acoustic rhinometry is an objective assessment of technique of nasal permeability that allows the determination of the transversal section of the area of any point between the nostril and rhinopharynx ^8^. Nasal volume between two points of the nasal cavity can also be calculated. The method is based on analysis of sound waves reflected by the nasal cavities in view of the sound stimulus. Incident and reflected nasal cavity sound waves are detected by a microphone and signals conducted to a computer program, which generates a graph of area as a result in distance (*area-distance curve* or *area-distance function*). The term nasal ecography is used by some authors ^9^, to avoid confusion between the terms rhinometry and rhinomanometry, which measures the airflow and intranasal pressure.

Based on the pioneer Danish study ^8^, the acoustic method started to be used in large scale in the study of nasal geometry and for the investigation of nasal physiological and pathological affections 1, 3. In addition not to being invasive, the method is quick and easy to be performed.

However, few studies apply acoustic rhinometry in the study of positional rhinitis 1, 3, 10. Fouke & Jackson in 1992^10^, O’Flynn in 1993^1^ and Kase et al. in 1994^3^, showed that acoustic rhinometry is a sensible technique to detect changes in nasal patency as a result of changes in body position.

Nasal resistance is mainly affected by the anterior nasal portion (nasal valve) where the narrowest segment is located. To assess it through rhinometry, a small change in the valve area causes exponential increase in nasal resistance and large affections in the posterior portion of the nasal cavity result in small changes of nasal resistance. According to the method of acoustic rhinometry, the area of cross section of any distance from the nostril and the nasal volume of any segment of the basal cavity can be directly measured ^3^. Thus, the present study aimed at:

a) Checking the effect of posture changes - seated and lying down positions - on nasal permeability in a group of normal subjects and in a group of subjects with clinical history of rhinitis using acoustic rhinometry.
b) Checking the effect of posture changes between seated and lying down positions on the perception of nasal breathing in a group of normal subjects and in a group of subjects with rhinitis using the visual analog scale.
c) Comparing the affections found in the two groups.

## MATERIAL AND METHOD

The study was conducted in the private office of the author in which the acoustic rhinometry device was located.

It is a transversal study in which the studied element was body posture affection and the outcome on nasal patency. We measured simultaneously the studied factor and the clinical outcome. The initial hypothesis was that rhinitic patients, upon changing positions (seated to lying down and also lying down to seated) would have higher likelihood of reducing nasal patency in comparison with normal patients.

Ten normal volunteers (6 men and 4 women) with ages between 19 and 30 years and 10 rhinitic patients with ages between 18 and 27 years (1 man and 9 women) were selected to the pilot study according to the pre-set inclusion and exclusion criteria. The participants were university students. We did not perform formal calculation of sample size because it was a pilot study.

We excluded from the study: normal participants with any acute nasal symptom during the week before and the day of the exam, normal subjects previously submitted to nasal or palate surgery, significant structural or mucosa affection detected in the anterior rhinoscopy, and rhinitis patients with chronic use of oral or topical antihistaminic, anticholinergic or corticoid decongestants (for the last month)

All participants responded to questionnaires and were submitted to anterior rhinoscopy executed by the main author. The person responsible for executing rhinometry did not know about the presence or not of rhinitic symptoms in the sample. All participants signed the Free Informed Consent Term. The project was submitted and approved by the Research Ethics Committee for Human and Animal Studies, Universidade Luterana do Brasil.

Acoustic rhinometry was preceded by a period of familiarization to the laboratory environment that lasted 30 minutes. After this period, we assessed nasal patency using an analog visual scale and patients were submitted to acoustic rhinometry in baseline conditions (seated position), then after 15 minutes in dorsal position and after 5 minutes back to the seated position.

**Figure 1:**
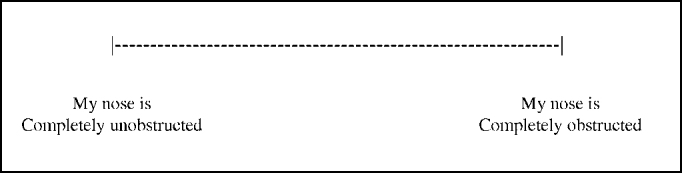
Visual analog scale.

The participants assessed global sensation of nasal permeability in a visual analog scale of 100mm of length ^11^ (Figure 1) in all three stages of survey (baseline seated, lying down, seated). The left extreme of the scale (0mm) corresponded to “my nose is completely unobstructed”, whereas the right extreme of the scale (100mm) was equivalent to “my nose is completely obstructed”.

After the record, they were submitted to acoustic rhinometry in seated positions, after 15 minutes in dorsal position and 5 minutes after they had reseated. The detailed protocol was the following:

I - adaptation
II - rating of seated position - visual analog scale (baseline)
III - seated acoustic rhinometry (baseline)
IV - 15 minutes lying down
V - rating of lying down - visual analog scale
VI - lying down acoustic rhinometry
VII - 5 minutes seated again
VIII - rating of seated position - visual analog scale
IX - seated acoustic rhinometry

**Figure 2:**
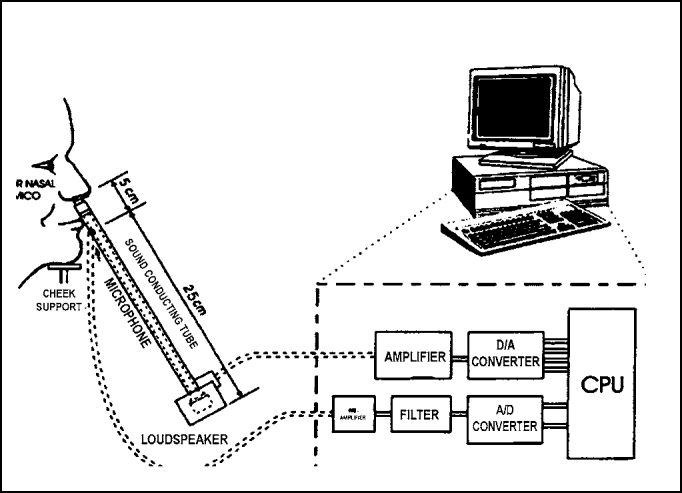
Diagram of acoustic rhinometry devices.

The devices (*Eccovision Acoustic Rhinometer - Model AR-1003, Hood Laboratories, Pembroke, MA*) constituted of a conducting sound tube of 30cm long to which a microphone was coupled in the proximal portion and a loudspeaker was coupled to the distal portion (Figure 2).

Pressure signals captured by the microphone were amplified and digitalized by a specifically installed board in an IBM computer, especially programmed for data recording and analysis. The program allows calculation of transversal section in any point of the nasal cavity from the nostril. Consequently, the nasal volume between two points could also be calculated.

A non-invasive nasal adapter was used to connect the sound conducting tube to the nostril to be tested. A sealing gel (*Eco Gel 400, Eco-Med Pharmaceuticals, Canada*) was applied to the borders of the nasal adapter to prevent air leak. The angle between the tube and the nasal floor was maintained at 45° during all measurements. Maximum area was taken to prevent air leak between the measurements and also to prevent nasal vestibule distortion.

The nasal cavities were tested separately. To each result, we considered the mean of 10 measurements by nasal fossa: (a) baseline conditions (seated position); (b) after 15 minutes in dorsal position; (c) after 5 minutes in seated position again. It is important to emphasize that each measurement takes about 10 seconds to perform and the time required to perform the whole protocol in one participant, after adaptation, was 20 minutes.

**Figure 3:**
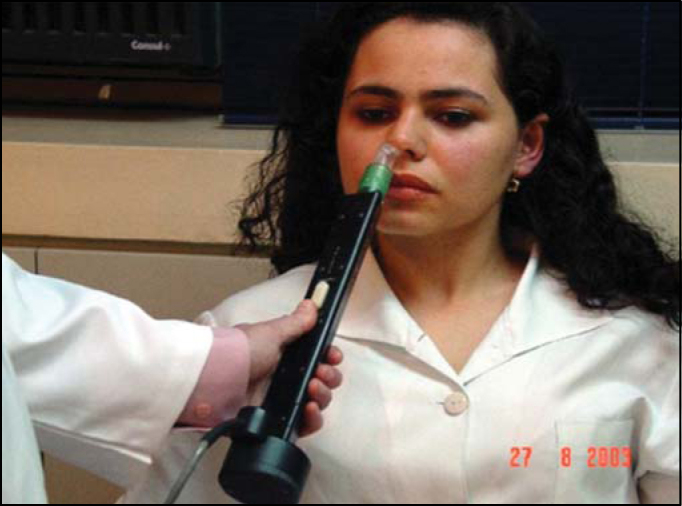
Volunteer seated during acoustic rhinometry.

**Figure 4:**
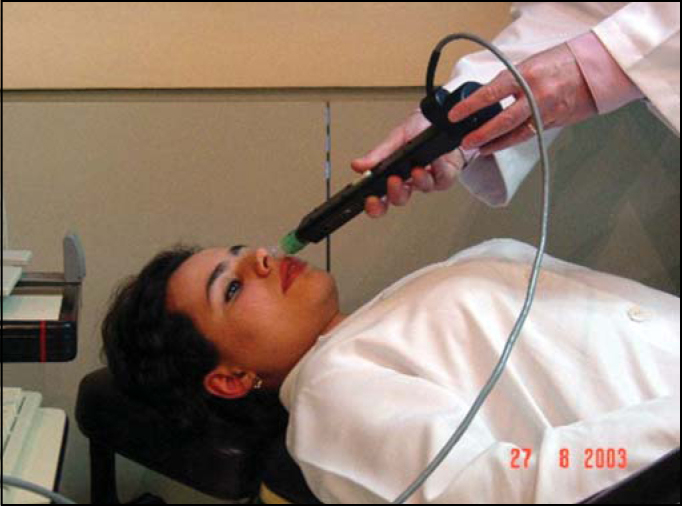
Volunteer in dorsal position during acoustic rhinometry.

Figures 3 and 4 illustrate a volunteer in the tested positions of the study.

To compare the means of each group (intra-group) we performed the t student test for paired samples. To compare differences of means between two groups (inter-group) we performed the test of variance, followed by t student test for independent samples. We considered significant differences as p<0.05.

## RESULTS

Table 1 describes the two groups, both normal and rhinitic groups, as a result of age, sex, weight and height.

**Table 1:** Description of the sample of normal and rhinitic patients.

	N	Age: interval	Sex: M/F	weight: mean (Kg)	height: mean (cm)
normal	10	19-30	6/4	63,75	169
rhinitic	10	18-27	1/9	59,50	167

Table 2 shows the findings of acoustic rhinometry in the group of normal and rhinitic patients in baseline conditions, after 15 minutes lying down and after 5 minutes again in seated position. Only total values of minimum transversal area and nasal volume are shown, that is, the adding up of nasal cavity values on the right and left.

**Table 2:** Total minimum transversal area and total nasal volume in baseline, lying down and sitting down positions.

	n	ATM total baseline	ATM total lying down	ATM total seated	VN total baseline	VN total lying down	VN total seated
normal	10	1,08±0,29	0,99±0,24	1,00±0,23	8,96±1,57	8,67±1,42	8,53±1,38
			*p<0,004			*p<0,01	
rhinitic	10	0,96±0,19	0,84±0,18	0,94±0,25	8,52±0,78	8,00±0,79	8,16±0,94
			*p<0,004	*p<0,02		*p<0,004	

mean ± standard deviation

ATM - total minimum transversal area (right + left) cm^2^

VN - total nasal volume (right + left) cm^3^

* p refers to ANOVA and paired T test compared to means in each group in positions baseline x lying down and lying down x seated.

Both groups (normal and rhinitic) show reduction of total minimum transversal area (p<0.004) and total nasal volume (p<0.01) when they went from initial seated position to lying down. When they sat again after 15 minutes lying down, only the total minimum transversal area in the rhinitic group showed significant improvement (p<0.02), restoring practically the value from baseline before lying down.

The reduction of area and total nasal volume noticed in the rhinitic group from initial seated position to lying down was not more marked than in the group of normal subjects (p=0.3 and p=0.4, respectively). Initial values of the minimum area and total nasal volume in seated position were not significantly different among subjects considered to be normal and those with clinical history of rhinitis. The perception of nasal obstruction was significantly higher in subjects with clinical history of rhinitis when compared to normal people (p=0.012).

Table 3 shows mean values of area and volume considering the nasal cavities of larger or smaller dimensions in the beginning of the study (initial seated position). In both the normal and the rhinitis groups, we observed reduction of area and nasal volume in both sides of the nose after 15 minutes in the lying down position. This reduction of area and volume, upon changing position from seated to lying down position, was higher in nasal cavities of smaller dimensions at initial baseline conditions.

**Table 3:** Minimum transversal area and nasal volume in positions baseline - lying down, considering the dimensions of nasal cavity.

	n	ATM small baseline	ATM small lying down	ATM large baseline	ATM large lying down	VN small baseline	VN small lying down	VN large baseline	VN large lying down
normal	10	0,46±0,16	0,38±0,13	0,61±0,14	0,60±0,15	4,13±0,80	3,89±0,64	4,83±0,82	4,78±0,82
			*p<0,001				*p<0,008		
rhinitic	10	0,41±0,11	0,32±0,11	0,56±0,11	0,51±0,10	3,97±0,43	3,66±0,48	4,55±0,44	4,34±0,52
			*p<0,001				*p<0,002		

mean ± standard deviation

ATM - minimum transversal area cm^2^

VN - nasal volume cm^3^

* p refers to Anova and paired t test compared to means in each group in positions baseline x lying down.

Table 4 shows values of nasal obstruction perception in both groups in the three positions, according to visual analog scale. The perception of nasal patency worsened significantly in normal (p<0.02) and rhinitis (p<0.05) subjects upon going from the initial seated position to lying down. Subjects with clinical history of rhinitis manifested worse perception of nasal breathing in relation to those without history, in all studied positions (p<0.01). The seated position after 15 minutes of lying down improved significantly the perception of nasal breathing in normal subjects (p<0.02), but not in the rhinitic ones (p=0.06).

**Table 4:** Perception of nasal obstruction in normal and rhinitic.

	n	EAV total baseline	EAV total lying down	EAV total seated
normal	10	0,84±0,80	1,54±1,16	0,86±0,80
			*p<0,02	*p<0,02
rhinitic	10	2,77±2,03	4,41±3,01	3,22±2,89
			*p<0,05	

mean ± standard deviation

EAV - visual analog scale

* p refers to ANOVA and t paired tests comparing means of each group in positions baseline x lying down and lying down x seated.

**Figure 5:**
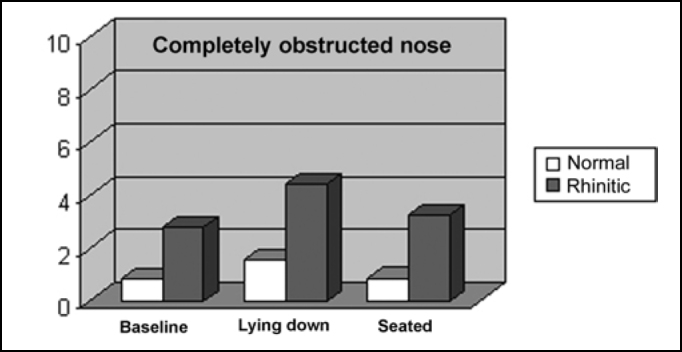
Perception of nasal breathing in the studied positions.

Figure 5 illustrates the perception of the two groups in positions assessed in the study.

## DISCUSSION

The present study showed that both normal and subjects with history of rhinitis presented worsening of nasal permeability when going from seated to lying down position. These findings were observed in the studied objective parameters (area and total nasal volume) and also in subjective parameters (perception of nasal breathing).

The effects of area and total nasal volume, observed by acoustic rhinometry, were similar in both groups, that is, regardless of the presence or not of rhinitis. However, subjects with rhinitis symptoms present higher subjective perception of nasal obstruction especially in dorsal position. We wondered whether the observation of this phenomenon would have two causes: first, because initial perception of nasal obstruction in the baseline was significantly higher than in subjects with history of rhinitis and, secondly, because patients with history of rhinitis normally present higher sensitivity to nonspecific stimuli ^13^. Previous studies demonstrated that inflamed nose, in which vascular tone is reduced in both nasal cavities, when on the lying down position will have significant worsening of nasal permeability 6, 14.

The restoration to seated position after 15 minutes in dorsal position was followed by significant improvement in perception of nasal breathing in the sample of normal subjects and it was almost significant in the sample of subjects with history of rhinitis (p<0.06). However, in the objective parameters, the total area of the subjects with history of rhinitis improved significantly after taking the seated position again. It may have resulted from the fact that the acoustic rhinometry was performed only 5 minutes after taking the seated position again. Another explanation may be lack of correlation demonstrated in other studies between findings of objective exams, such as nasal rhinoscopy and acoustic rhinometry, and perception of breathing ^15^. Moreover, Kase et al. ^3^ showed that the minimum area seems to be more sensible than volume to detect posture changes. These observations confirm the clinical recommendation of elevating the bed top, normally used in patients with decubitus rhinitis.

Another relevant factor is that baseline conditions of acoustic rhinometry values for minimum area and total nasal volume were not statistically significant different between normal and rhinitic subjects. The subjective perception of nasal obstruction, in turn, was much enhanced in the group with rhinitis. It is in agreement with the literature that shows that acoustic rhinometry, as well as other methods to test nasal patency, may not be capable of differentiating normal from abnormal noses, and it is very important to consider the clinical history ^16^. Another factor is the criterion adopted for the sample of rhinitic patients in this study: the clinical history, in which we did not reach diagnostic laboratory confirmation (ex: measurement of IgE).

Studies that used rhinomanometry as objective method of measurement of nasal permeability showed significant reduction of transnasal airflow in the most closed cavity, when the patient takes the supine position 4, 5. Even so, when lying down on lateral position, the side of the patients’ nose that is down is worse and the other nostril improves its permeability. The opposite happens when the position is inverted. Cole & Haight^2^, however, noticed that in normal subjects total nasal resistance remains sort of constant. In rhinitis patients, total nasal resistance was decreased in lying down position 6, 14.

The results of our study in relation to influence of posture over nasal permeability measured by acoustic rhinometry are in agreement with the few previous studies already performed.

Fouke & Jackson^10^ used acoustic rhinometry in 8 normal subjects and described that 15 minutes after the body had been turned to supine position to the right side, ipsilateral nasal volume reduced significantly, from about 29.3±4.4 to 19.5±3.6 cm^3^ (p<0.003) and contralateral nasal volume increased significantly from 20.9±2.8 to 25.5±3.2 cm^3^ (p<0.05). The purpose of our study was not to analyze the influence of lateral position over nasal permeability, given that it is quite difficult to manage sealing of the nasal cavity that is on the lower position to appropriately perform acoustic rhinometry.

Kase et al. ^3^ showed 8 young adult subjects that 6 minutes after going from seated to lying down position had total dimension of the airway (sum of minimum transversal area on both sides) reduced by about 16%. Moreover, the study showed that measurement of the minimum area was more sensible than that of nasal volume for the detection of posture affections. Our findings in normal and rhinitic subjects show a reduction in total minimum transversal area of 9% or 12.5%, respectively.

In addition, Kase et al. ^3^ noticed that despite the fact that the change in seated position to lying down position caused narrowing of nasal cavities, the same was only significant in the narrower side. Considering the narrow side, the minimum transversal area reduced approximately 20% and nasal volume was reduced 10%. Our study also showed that the closest side in initial seated position (baseline) presented the most significant change 15 minutes after taking on dorsal position, and the values were 17.7% for the area and 5.8% for volume in normal subjects. Rhinitis subjects had reduction of area which was more significant in the nasal cavity initially in the closet position (reduction of 22%) when compared to that that occurred in the initially opened side (reduction of 8.9%).

O’Flynn^1^ showed the opposite in 14 normal young adults, that is, the changes were more marked on the nose side that was patent in the initially seated position. Moreover, a discreet increase in volume in the closed cavity in the seated position was observed after 5 minutes of lying down. The study revealed a reduction in minimum area when going from seated to lying down position, but it was not statistically significant. However, in this study, subjects remained lying down for only 5 minutes, differently from our study in which they remained 15 minutes in dorsal position. Studies that used rhinomanometry ^7^, such as in our study and the study by Kase et al. ^3^, showed that the effect of dorsal position is very much marked in the nasal cavity that is initially closed or less permeable.

In summary, our study using acoustic rhinometry confirms the already known effect of dorsal position over nasal permeability, that is, its reduction when going from seated position to lying down position. It was not object of the present study to reveal the mechanisms responsible for this fact, but both local factors (congestion of capacitance vessels of inferior concha mucosa) and systemic ones (increase in venous pressure by the affection of hydrostatic pressure), both related to cutaneous and vascular reflexes, seem to be involved.

## CONCLUSION

a) Posture affections between the seated and lying down positions worsen nasal permeability, with reduction of minimum area and total nasal volume, both in normal and in rhinitic subjects.
b) The worsening in minimum area and in total nasal volume observed in dorsal position is followed by worsening of nasal breathing perception, both in normal and in rhinitic subjects.
c) The perception of nasal obstruction as a result of posture affections is higher in subjects with history of rhinitis than in normal subjects.
